# Antimicrobial Resistance in Swine Fecal Specimens Across Different Farm Management Systems

**DOI:** 10.3389/fmicb.2020.01238

**Published:** 2020-06-17

**Authors:** Suporn Pholwat, Tawat Pongpan, Rattapha Chinli, Elizabeth T. Rogawski McQuade, Iyarit Thaipisuttikul, Parntep Ratanakorn, Jie Liu, Mami Taniuchi, Eric R. Houpt, Suporn Foongladda

**Affiliations:** ^1^Department of Microbiology, Faculty of Medicine Siriraj Hospital, Mahidol University, Bangkok, Thailand; ^2^Division of Infectious Diseases and International Health, Department of Medicine, University of Virginia, Charlottesville, VA, United States; ^3^Swine Veterinarian Service, Charoen Pokphand Foods PCL, Bangkok, Thailand; ^4^Faculty of Veterinary Science, Mahidol University, Nakhon Pathom, Thailand

**Keywords:** antimicrobial resistance, AMR, swine, farm management, fecal specimens

## Abstract

Antimicrobial use in agricultural animals is known to be associated with increases in antimicrobial resistance. Most prior studies have utilized culture and susceptibility testing of select organisms to document these phenomena. In this study we aimed to detect 66 antimicrobial resistance (AMR) genes for 10 antimicrobial agent classes directly in swine fecal samples using our previously developed antimicrobial resistance TaqMan array card (AMR-TAC) across three different swine farm management systems. This included 38 extensive antimicrobial use (both in treatment and feed), 30 limited antimicrobial use (treatment only), and 30 no antimicrobial use farms. The number of resistance genes detected in extensive antimicrobial use farms was higher than in limited and no antimicrobial use farms (28.2 genes ± 4.2 vs. 24.0 genes ± 4.1 and 22.8 genes ± 3.6, respectively, *p* < 0.05). A principal component analysis and hierarchical clustering of the AMR gene data showed the extensive use farm samples were disparate from the limited and no antimicrobial use farms. The prevalence of resistance genes in extensive use farms was significantly higher than the other farm categories for 18 resistance genes including *bla*_SHV_, *bla*_CTX–M1_ group, *bla*_CTX–M9_ group, *bla*_VEB_, *bla*_CMY2–LAT,_ *aac(6′)-lb-cr*, *qnrB1*, *gyrA*83L-*E. coli*, *armA*, *rmtB*, *aac(3)-IIa, mphA*, 23S *rRNA* 2075G-*Campylobacter* spp., *mcr-1*, *catA1*, *floR*, *dfrA5-14*, and *dfrA17*. These genotypic findings were supported by phenotypic susceptibility results on fecal *E. coli* isolates. To examine the timing of AMR gene abundance in swine farms, we also performed a longitudinal study in pigs. The results showed that AMR prevalence occurred both early, presumably from mothers, as well as after weaning, presumably from the environment. In summary, detection of AMR genes directly in fecal samples can be used to qualitatively and quantitatively monitor AMR in swine farms.

## Introduction

Antimicrobial resistance (AMR) in bacteria is driven by the selective pressure of antibiotics and exerts an enormous disease burden and increased economic costs (Founou et al., 2017). More than half of all antimicrobial use occurs in the agricultural industry for treatment or as feed additives for infection prophylaxis and growth promotion (Neill, 2015). Economic development and population growth have increased demand for animal protein and resulted in a burgeoning agricultural production industry (Yunhe Cao, 2013; Walther et al., 2016). While the use of antimicrobial agents as feed additives is prohibited in some countries, it is commonly practiced in many Southeast Asian countries (Nhung et al., 2016; Zellweger et al., 2017). This overuse of antimicrobial agents in livestock is one likely driver of the high AMR burden in Southeast Asia, including high rates of extended spectrum β-lactamase (ESBL) and CTX-M enzymes (Zellweger et al., 2017). AMR in food animals can impact human health by the direct introduction of AMR pathogens into the food chain, by promoting horizontal transfer of resistance determinants to other bacterial pathogens (Munita and Arias, 2016), and by indirect spread to other animals and humans via water and soil (Marshall and Levy, 2011; Woolhouse et al., 2015).

Recently, no antimicrobial use farms have increased and studies have investigated if these farms decrease occurrence of AMR as measured by standard culture and phenotypic antimicrobial susceptibility testing (AST) of a sentinel bacterial species (Luangtongkum et al., 2006; Lestari et al., 2009; Osterberg et al., 2016; Kempf et al., 2017; Lugsomya et al., 2018a,b). These studies have demonstrated that stopping use of antimicrobials in feed has been associated with decreased rates of AMR.

However, culture of fecal organisms is laborious and inherently selective. Therefore in this study we measured AMR genes directly in fecal specimens using our previously developed TaqMan array card (Pholwat et al., 2019) which simultaneously detects 66 resistance-associated genes or mutations to 10 antimicrobial agent classes commonly used in human and veterinary medicine including penicillins, cephalosporins, carbapenems, fluoroquinolones, macrolides, aminoglycosides, folate pathway inhibitors, tetracyclines, phenicols, and polymyxins. The assays were largely directed toward Enterobacteriaceae, the leading AMR bacterial family in the intestinal tract. We focused the study on swine in Thailand, where pork is the second most produced meat after chicken (Coyne et al., 2019), since some reviews have indicated a higher probability of AMR in pigs than in chicken, other food animals, or aquaculture (Nhung et al., 2016).

## Materials and Methods

### Study Design

The primary objective was to perform a cross-sectional study to examine three different swine management systems including extensive antimicrobial use (treatment and feed), limited antimicrobial use (treatment only), and no antimicrobial use farms. The antimicrobial agents used in each group are detailed in Table 1. Our minimum sample size requirement was 100 pigs for each group. The sampling method was a simple convenience sampling of five pens per farm and the unit of analysis was per sample. The secondary aim was a longitudinal study to determine when AMR prevalence or acquisition occurred during the production stages by measuring AMR in sows, their 3-week old weaning piglets, and at the 24-week old finishing stage.

**TABLE 1:** Antimicrobial agent exposure in 6 months of raise for each farm categories.

Purpose of use	Antimicrobial classes	Antimicrobial agents	Farm categories		
			Extensive antimicrobial use	Limited antimicrobial use	No antimicrobial use
Feed additive	β-lactam	Amoxicillin	+	−	−
	Macrolides	Kitasamycin, tilmicosin, tylosin, tylvalosin	+	−	−
Treatment^a^	β-lactam	Amoxicillin, ampicillin, penicillin, ceftiofur	+	+	−
	Fluoroquinolones	Enrofloxacin, marbofloxacin	+	+	−
	Aminoglycosides	Gentamicin, neomycin, streptomycin	+	+	−
	Polymyxins	Colistin	+	−	−

*+ indicate use those antimicrobial classes, − indicate not use those antimicrobial classes. ^a^Not all farms used the same antimicrobial agents and may not use all antimicrobial agents list, for instance farm A used β-lactam, fluoroquinolones, aminoglycosides, and polymyxins for treatment while farm B used only β-lactam and aminoglycosides.*

### Enrolled Farms

All farms enrolled in this study were contracted or belong to a commercial company which practices the three different farm management systems. Ninety-eight swine farms located in 25 provinces in Thailand were enrolled into the primary study based on those where pigs aged 20–24 weeks were available and the owner agreed to participate the study. These included 38 extensive antimicrobial use farms (Ex-f) located in Lopburi, Nakhon Pathom, Saraburi (central), Chonburi, Chanthaburi, Rayong (eastern), Kanchanaburi (western), Chaiyaphum, Nakhon Phanom, Nong Bua Lam Phu, Udon Thani (northeastern), Nakhon Sawan, Phetchabun, Phitsanulok, Phrae, and Uttaradit (northern) provinces. For limited antimicrobial use farms (Li-f) and no antimicrobial use farms (No-f), we included 30 farms for each group and all farms located in northern part of Thailand including Chiang Mai, Chiang Rai, Lumpang, Lamphun, Nan, Nakhon Sawan, Phetchabun, Phichit, Phitsanulok, Phrae, SukhoThai, Tak, and Uttaradit provinces (Supplementary Figure S1).

The farms enrolled in the primary study were fattening farms which received a weaned piglet from other independent breeding farms. The capacity of farms ranged between 400 and 800 pigs and used an all-in all-out production system. For Ex-f, combinations of amoxicillin, kitasamycin, tilmicosin, tylosin, tylvalosin, tiamulin, and halquinol such as amoxicillin+halquinol+tilmicosin, amoxicillin+halquinol, amoxicillin+tiamulin, or amoxicillin+tilmicosin were routinely mixed into the feed at a concentration ranging between 100 and 300 part per million (ppm) during the growing periods. The Ex-f had consistent antimicrobial use in-feed for at least 2 years. For Li-f, antimicrobial agents were used at least once during the 6-month production period when animals became ill, whereupon antibiotics were administered through oral solution and/or injection with blanket treatment of the farm. Five farms were enrolled into a secondary study including 1 breeding farm located in Phetchabun province and four fattening farms located in Phetchabun and Saraburi provinces, all of which were Ex-f.

### Specimen Collection

For the primary study, the first 5 fresh fecal samples were collected from the floor of 5 pens of healthy 20–24-week old finishing pigs from each farm between February 2018 and February 2019 yielding 490 samples. For the secondary study, paired rectal swabs from sows (*n* = 14) and their 3-week old weaning piglets (*n* = 25) were collected from breeding farms in February 2019, then piglets were ear tagged and moved to fattening farms where the follow-up 24 weeks old samples were collected from the same pigs between July and August 2019. Fecal and rectal swab samples were collected by local veterinary assistants. Fecal specimens were collected and transported in a sterile container while rectal swabs were collected and transported in PrimeStore molecular transport medium (Longhorn Vaccines and Diagnostics LLC, Antonio, TX, United States) and FecalSwab (COPAN Diagnostics Inc., Murrieta, CA, United States). Specimens were transported to the laboratory under cold chain and stored at −70°C for future specimen processing. For culture, fecal samples and rectal swabs were streaked on CHROMagar *E. coli* (DRG International Inc., Springfield, NJ, United States) and incubated at 35 ± 2°C for 18–24 h. Five to ten *E. coli* colonies were pooled and stored in preservative media at −70°C. This animal specimen collection protocol no. 013/2561 was reviewed and approved by Siriraj Animal Care and Use Committees, Faculty of Medicine Siriraj Hospital, Mahidol University.

### DNA Extraction

Genomic DNA from fecal and rectal swab specimens was extracted using the QIAamp Fast DNA Stool mini kit (Qiagen, Valencia, CA, United States). Briefly, 200 mg or 200 μl of specimen was suspended with 1 ml InhibitEX buffer and incubated at 95°C for 5 min followed by centrifugation at 20,000 × *g* for 1 min. Supernatant (600 μl) was transferred to a new tube containing 25 μl proteinase K followed by 600 μl lysis buffer AL then mixed and incubated at 70°C for 10 min. Six hundred μl of ethanol was added into lysate and mixed rigorously. The lysate was then purified through QIAamp mini spin column following the manufacturer’s instructions, eluted into 200 μl, and the eluate was stored at −20°C to be used as DNA template.

### Antimicrobial Resistance Genes Detection

Our custom-developed antimicrobial resistance TaqMan array card (AMR-TAC) (Applied Biosystems, Thermo Fisher Scientific, Foster City, CA, United States) was utilized as previously described (Pholwat et al., 2019). Briefly, primer and TaqMan probe oligonucleotides were synthesized and spotted onto the microfluidic card. Twenty microliters of input DNA was mixed with 50 μl of 2x PCR buffer, 4 μl of 25× PCR enzyme of AgPath-ID-PCR kit (Applied Biosystems, Life Technologies Corporation), and 26 μl of nuclease free water to yield a 100 μl final volume. This mixture was loaded into each port of the card and the card was centrifuged twice at 1,200 rpm for 1 min and then sealed. The loading ports were excised and the full card was inserted into a ViiA 7 instrument (Applied Biosystems, Life Technologies Corporation). Cycling conditions included an initial denaturation at 95°C for 10 min, followed by 40 cycles of denaturation at 95°C for 15 s and annealing/extension at 60°C for 1 min.

### Standard Curve and Normalization

Synthetic positive control plasmids (Genewiz Inc., South Plainfield, NJ, United States) which contained primer/probe regions of all targets were 10-fold serially diluted in a range of 5 × 10^7^ to 5 copy/μl. Twenty microliters of each diluted sample was tested in triplicate by mixing with PCR reagents to a total 100 μl then loaded into the array card. The final concentration ranged from 1 × 10^7^ to 1 copy/reaction. The average cycle threshold (Ct) value was used to generate standard curve for later quantification and conversion of Ct values to gene copy number of fecal specimens. The Ct value was converted to copy number by using standard curves for each target and then the resistance gene copy number was normalized to 10^6^ bacterial 16S *rRNA* gene copy number of each sample. These yielded copies of resistance genes per 10^6^ bacterial 16S *rRNA* gene copies, which allowed comparisons between fecal and swab specimens. The normalized gene copy number (gene copy) was used for analysis and when binary (positive/negative) results were required, the gene copy cut-off was applied whereby ≥ 1 gene copy was positive.

### Phenotypic Antimicrobial Susceptibility Testing

Two hundred and forty-six *E. coli* isolates were randomly selected from a total of 490 isolates from fecal samples collected from three different farm categories for phenotypic AST. Prior to susceptibility testing, *E. coli* isolates were subcultured on blood agar (TSA w/5% sheep blood, Thermo Scientific, NY, United States) at 35 ± 2°C for 18–24 h. The *E. coli* isolates underwent susceptibility testing by disk diffusion for ampicillin (AMP; 10 μg), amoxicillin-clavulanic acid (AmC; 20:10 μg), cefazolin (CFZ; 30 μg), cefoxitin (FOX; 30 μg), ceftazidime (CAZ; 30 μg), ceftiofur (XNL; 30 μg), ceftriaxone (CRO, 30 μg), imipenem (IPM; 10 μg), azithromycin (AZM; 15 μg), nalidixic acid (NA; 30 μg), ciprofloxacin (CIP; 5 μg), enrofloxacin (ENO; 5 μg), amikacin (AMK; 30 μg), gentamicin (GM; 10 μg), kanamycin (KAN; 30 μg), streptomycin (STR; 10 μg), trimethoprim/sulfamethoxazole (TMP-SMX; 1.25/23.75 μg), tetracycline (TET; 30 μg), and chloramphenicol (CHL; 30 μg). The broth microdilution method was used for florfenicol (FLO) and colistin (CL). All antimicrobial disks were obtained from Becton Dickinson (Becton Dickinson, Sparks, MD, United States) except AZM, AMK, and CFZ were obtained from Oxoid (Oxoid Limited, Hampshire, United Kingdom) and FLO and CL powder were obtained from Sigma (Sigma-Aldrich, St. Louis, MO, United States).

The methodologies were performed according to the Clinical and Laboratory Standards Institute (CLSI, 2018d,a,b) as described previously (Pholwat et al., 2019). Briefly, for disk diffusion, bacterial suspensions were prepared in normal saline and adjusted to 0.5 McFarland standards then suspensions were dipped by sterile cotton swab and swabs were streaked over the entire Mueller Hinton agar (MHA, BD Difco Mueller Hinton Agar, Becton Dickinson) surface. Disks containing antimicrobials were placed onto the surface of the inoculated agar plate and incubated at 35 ± 2°C for 16–18 h. For broth microdilution, antimicrobial agents were 2-fold serially diluted in cation-adjusted Mueller Hinton broth (CAMHB, BD BBL Mueller Hinton II Broth, Becton Dickinson) and 100 μl of each dilution including no-antimicrobial control media were dispensed into 96 well round bottom culture plates. The bacterial suspensions (0.5 McFarland) were diluted at 1:20 in cation-adjusted Mueller Hinton broth to obtain 5 × 10^6^ cfu/ml. Then 10 μl of bacterial inoculum was inoculated into 96 well round bottom plates and incubated at 35 ± 2°C for 16–20 h. *E. coli* ATCC 25922, *P. aeruginosa* ATCC 27853 (for carbapenem), and *S. aureus* ATCC 25923 (for azithromycin) were used as quality control and the minimal inhibitory concentration (MIC) and zone diameter interpretative standards of CLSI-M100 Ed29 and CLSI-VET08 Ed4 (CLSI, 2018c, 2019) were used for interpretation. Intermediate was considered resistant in this study.

### Statistical Analysis

The principal component analysis and hierarchical clustering were generated using ClustVis https://biit.cs.ut.ee/clustvis/ (Metsalu and Vilo, 2015). The singular value decomposition (SVD) with imputation was used to calculate principal components and Euclidean distance and average linkage were used for clustering. We used a permutational multivariate analysis of variance (PERMANOVA) test to test differences in antimicrobial resistance genes between antimicrobial use groups by using the Adonis function of the vegan package in R version 3.6.3. To account for clustering of samples within farms, we used a generalized linear mixed model for the first principal component and included farm as a random effect. For the longitudinal study, in addition to farm the family (sows, 3-week old, and 24-week old pig) was included as a random effect to account for clustering within family. One-way ANOVA, Chi-square test, Fisher’s exact test, Kruskal-Wallis *H* test, and Friedman test were performed with IBM SPSS Statistics Software version 26. The Benjamini-Hochberg procedure was used to control the false discovery rate at 5% for multiple comparisons.

## Results

### AMR Genes Among Three Different Farm Categories

The samples were from 38 extensive antimicrobial use farms (Ex-f) (*n* = 190), 30 limited antimicrobial use farms (Li-f) (*n* = 150), and 30 no antimicrobial use farms (No-f) (*n* = 150). There was no significant difference in the age of sampled pigs among the farms: Ex-f (23.9 ± 1.9 weeks), Li-f (23.8 ± 1.0), and No-f (23.6 ± 0.8, *p* > 0.05; One-way ANOVA).

**FIGURE 1:**
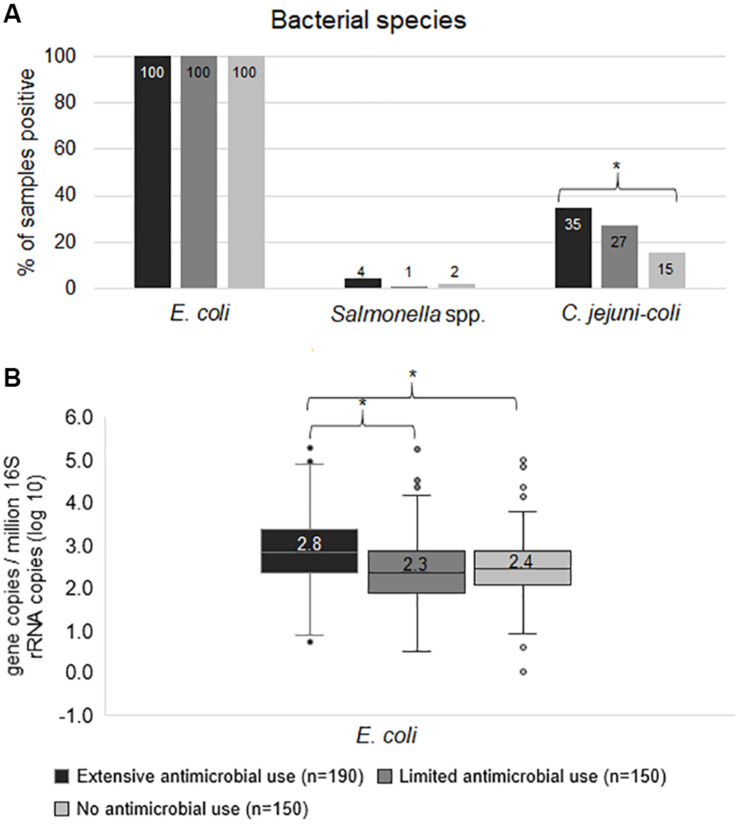
Frequency of bacterial species. The prevalence of bacterial species **(A)** and quantity of *E. coli* **(B)** among extensive antimicrobial use, limited antimicrobial use, and no antimicrobial use farms. The gene copy is shown whereby ≥ 1 is positive. For quantity of *E. coli* **(B)**, the gene copies were plotted. The horizontal line within the box indicates median, boundaries of the box indicate the 25th and 75th percentile, the whiskers indicate the highest and lowest values, the circles (o) indicate outliers. Asterisk symbol (*) indicate statistically significance (*p* < 0.05, Chi-square test or Kruskal-Wallis *H* test).

In terms of bacterial species, *E. coli* was positive in 100% of samples, however, the median gene copy of *E. coli* in Ex-f (log_10_ = 2.8) was higher (*p* < 0.05, Kruskal-Wallis *H* test) than in Li-f (log_10_ = 2.3) and No-f (log_10_ = 2.4) (Figures 1A,B). Additionally, the linear mixed model accounting for clustering within farm confirmed significant difference between Ex-f and Li-f as well as between Ex-f and No-f (*p* < 0.001). The prevalence of *Salmonella* spp. was 4, 1 and 2% and *C. jejuni-coli* was 35, 27, and 15% for Ex-f, Li-f and No-f, respectively (Figure 1A).

**FIGURE 2:**
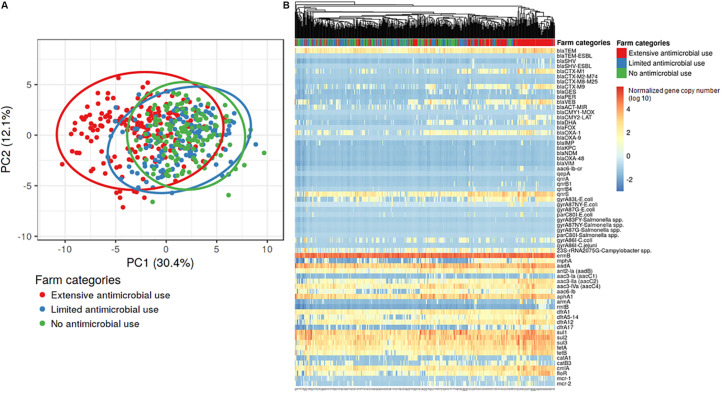
Principal component analysis and hierarchical clustering of AMR genes. The gene copies of samples from the three different farm categories were subjected to principal component analysis **(A)** and hierarchical clustering **(B)**. Singular value decomposition (SVD) with imputation was used to calculate principal components. The *X* and *Y* axis showed principal component 1 and principal component 2 that explain 30.4 and 12.1% of the total variance, respectively. Prediction ellipses were such that a new observation from the same group will fall inside the ellipse at a probability of 0.95, *N* = 490 data points. For hierarchical clustering **(B)**, no scaling is applied to rows and columns are clustered using Euclidean distance and average linkage. 66 rows represent each resistance gene and 490 columns represent each sample.

The normalized copy numbers of 66 AMR genes were subjected to hierarchical clustering and principal component analysis (PCA). Principal component analysis of the AMR gene data showed some overlap among the three farm categories, however, the Ex-f was generally quite different from the Li-f and No-f (Figure 2A). Hierarchical clustering showed that most Ex-f samples clustered together (Figure 2B), and the heat map of gene copies visually suggested higher quantities of certain AMR genes such as *bla*_CTX–M1_ group, *bla*_CTX–M9_ group, and *bla*_OXA–1_ in these farms. There was a significant difference in AMR genes between Ex-f and No-f, between Ex-f and Li-f (*p* < 0.001, PERMANOVA test and *p* < 0.001, linear mixed model accounting for clustering within farm), as well as between Li-f and No-f (*p* = 0.03, PERMANOVA test and *p* = 0.03, linear mixed model).

### Genotypic Antimicrobial Resistance Prevalence

We then sought to examine more directly the specific genes that explained the difference in the Ex-f. Sixty-six resistance associated genes or mutations were tested. This showed that the number of resistance genes in Ex-f (28.2 ± 4.2) was higher than Li-f (24.0 ± 4.1) and No-f (22.8 ± 3.6) (*p* < 0.05, One-way ANOVA).

**FIGURE 3:**
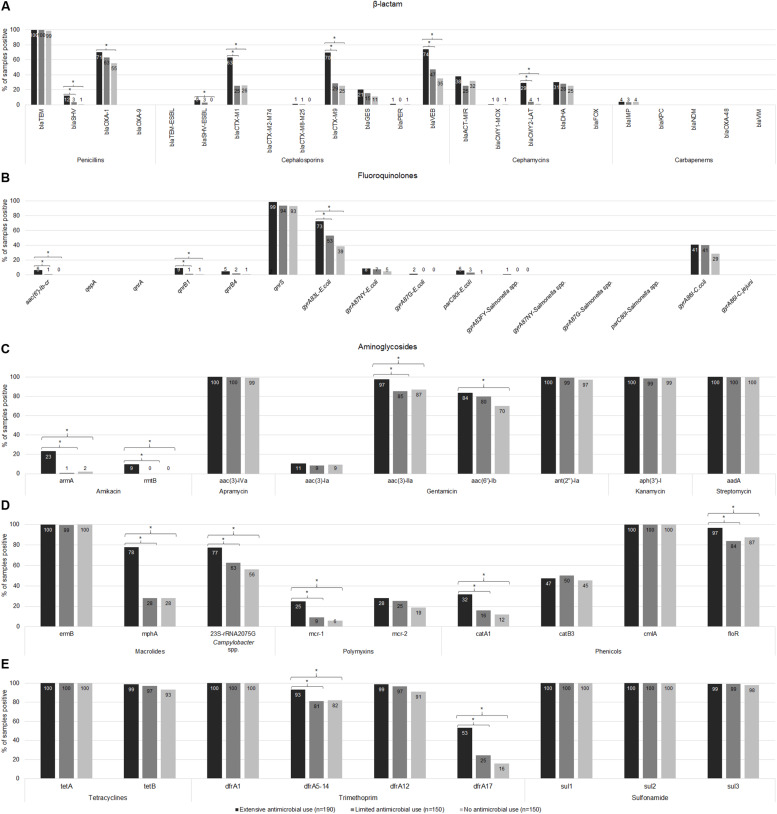
Prevalence of AMR genes. The gene copy is shown whereby ≥ 1 is positive. The prevalence of resistance-associated genes or mutations of β-lactam **(A)**, fluoroquinolones **(B)**, aminoglycosides **(C)**, macrolides, polymyxins, and phenicols **(D)**, tetracyclines and trimethoprim-sulfonamides **(E)** among extensive antimicrobial use, limited antimicrobial use, and no antimicrobial use farms were shown. Asterisk symbol (*) indicated statistically significant (*p* < 0.05, Chi-square or Fisher’s exact test) different of proportion between groups under the brace.

The prevalence of resistance genes in the Ex-f was higher (*p* < 0.05, Chi-Square or Fisher’s Exact) than Li-f and No-f for 18 (27%) resistance genes such as *bla*_SHV_, *bla*_CTX–M1_ group, *bla*_CTX–M9_ group, *bla*_VEB_, and *bla*_CMY2–LAT_ for β-lactam (Figure 3A); *aac(6′)-lb-cr*, *qnrB1*, and *gyrA*83L-*E. coli* for fluoroquinolones (Figure 3B); *armA*, *rmtB*, and *aac(3)-IIa* for aminoglycosides (Figure 3C); *mphA* and 23S *rRNA* 2075G-*Campylobacter* spp. for macrolides; *mcr-1* for polymyxins *catA1* and *floR* for phenicols (Figure 3D); *dfrA5-14* and *dfrA17* for trimethoprim (Figure 3E). There was no difference between Ex-f and Li-f but there was a difference between Ex-f and No-f for *bla*_OXA–1_, *bla*_SHV–ESBL_ (Figure 3A), and *aac(6′)-Ib* (Figure 3C).

Fourteen (21%) resistance genes were negative in all samples including *bla*_OXA–9_, *bla*_TEM–ESBL_, *bla*_CTX–M2–M74_ group, *bla*_FOX_, *bla*_KPC_, *bla*_NDM_, *bla*_OXA–48_, and *bla*_VIM_ for β-lactam (Figure 3A); *qepA*, *qnrA*, *gyrA*87NY-*Salmonella* spp., *gyrA*87G-*Salmonella* spp., *parC*80I-*Salmonella* spp., and *gyrA*86I-*C. jejuni* for fluoroquinolones (Figure 3B). The other 31 (47%) AMR genes did not differ in prevalence among the three farm categories and 15 (23%) of these genes were highly prevalent in all farms (91%–100%) including *bla*_TEM_ (Figure 3A), *qnrS* (Figure 3B), *aac(3)-IVa*, *ant(2″)-Ia*, *aph(3′)-I*, and *aadA* (Figure 3C), *ermB* and *cmlA* (Figure 3D), *tetA*, *tetB*, *dfrA1*, *dfrA12*, *sul1*, *sul2*, and *sul3* (Figure 3E).

**FIGURE 4:**
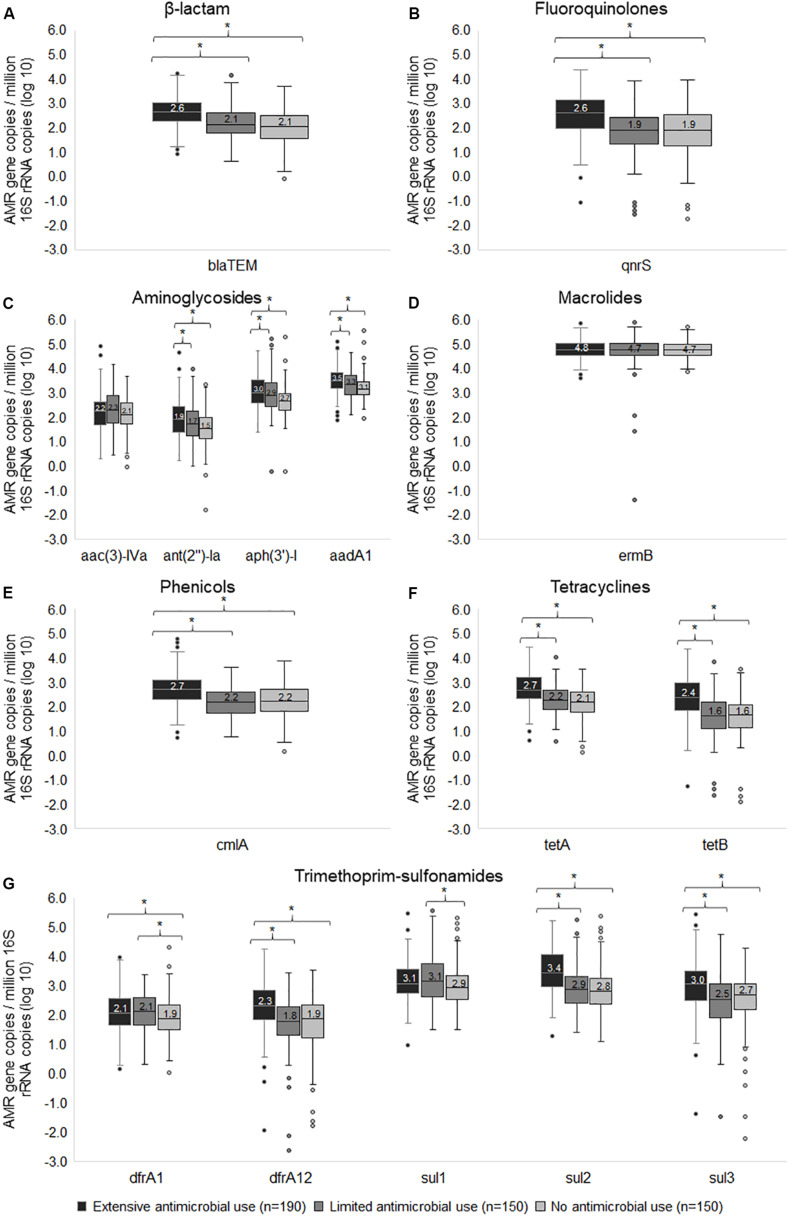
Box and whisker plots of wide-spread resistance genes. The normalized gene copy number of resistance genes associated β-lactam **(A)**, fluoroquinolones **(B)**, aminoglycosides **(C)**, macrolides **(D)**, phenicols **(E)**, tetracyclines **(F)**, and trimethoprim-sulfonamides **(G)** among extensive antimicrobial use, limited antimicrobial use, and no antimicrobial use farms were plotted. The horizontal line within the box indicates median, boundaries of the box indicate the 25th and 75th percentile, the whiskers indicate the highest and lowest values, the circle (o) indicates outlier. *, indicates *p* < 0.05, Kruskal-Wallis *H* test between medians of groups.

In addition to detecting gene prevalence, we examined the gene copies to assess quantity. The median gene copies in Ex-f was significantly higher (*p* < 0.05, Kruskal-Wallis *H* test) than Li-f and No-f for 11 resistance genes including *bla*_TEM_ for β-lactam (Figure 4A); *qnrS* for fluoroquinolones (Figure 4B); *ant(2″)-Ia*, *aph(3′)-I* and *aadA* for aminoglycosides (Figure 4C); *cmlA* for phenicols (Figure 4E); *tetA* and *tetB* for tetracyclines (Figure 4F); and *dfrA12*, *sul2* and *sul3* for trimethoprim-sulfonamides (Figure 4G). There was also a difference between Li-f and No-f for *dfrA1* and *sul1* (Figure 4G). Finally, two resistance genes that were not significantly different even in quantity among three farm categories included *aac(3)-IVa* for aminoglycosides (Figure 4C) and *ermB* for macrolides (Figure 4D). The linear mixed model was performed to account for clustering within farms, and there remained a significant difference in quantity of genes between Ex-f and No-f and between Ex-f and Li-f (*p* < 0.001) for *bla*_TEM_, *qnrS*, *dfrA12*, *sul2*, *sul3*, *tetA*, *tetB*, and *cmlA*. There was also a significant difference between Ex-f and No-f (*p* < 0.05) but no significant difference between Ex-f and Li-f for *aadA*, *ant(2″)-Ia*, and *aph(3′)-I*, and no significant difference in quantity of genes among the three farm categories for *dfrA1*, *sul1*, *ermB*, and *aac(3)-IVa*.

Among beta-lactam resistance detected genes, 15/23 genes were positive as shown in Figure 3A. The frequency of positive samples in Ex-f (*n* = 190) was 74.2% for *bla*_VEB_, 70.0% for *bla*_CTX–M9_ group, and 63.2% for *bla*_CTX–M1_ group, whereas Li-f (*n* = 150) was 47.3, 28.7, and 25.3%, respectively; and No-f (*n* = 150) was 35.3, 25.3, and 26.0%, respectively. Although, *bla*_OXA–1_ positive was highest in Ex-f (70.5%), the prevalence of this gene was also high in Li-f (63.3%) and in No-f (55.3%). The *bla*_SHV_ was found in 12.1% of Ex-f samples and half (6.3%) was the common amino acid substitution Gly238, and Glu240 which is *bla*_SHV–ESBL_. In Li-f, *bla*_SHV_ was presented in only 5 samples (3.3%) and 4 of which (2.7%) was *bla*_SHV–ESBL_. Only one sample in No-f contain *bla*_SHV_.

For *bla*_CMY2–LAT_, 29.5% of samples were positive in Ex-f while Li-f and No-f were only 4.0 and 1.3% positive, respectively. No significant difference of *bla*_DHA_ frequency was observed among Ex-f (30.5%), Li-f (28.0 %) and No-f (25.3%). Although, *bla*_GES_ presented in 20.5% of Ex-f, 15.3% of Li-f and 11.3% of No-f, they were not significantly different (*p* = 0.069). Almost all samples had *bla*_TEM_, however, Ex-f had a higher median copy number (log_10_ = 2.6) than Li-f (log_10_ = 2.1) and No-f (log_10_ = 2.1; Kruskal-Wallis *H* test), as shown in Figure 4A.

**FIGURE 5:**
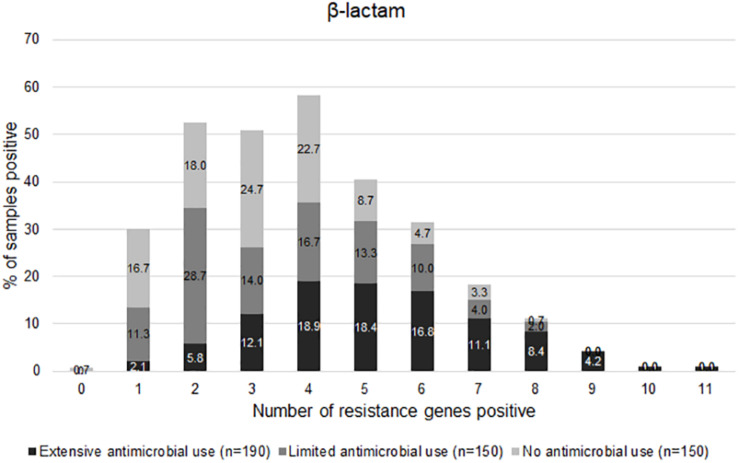
Distribution of AMR genes positive. The normalized gene copy number is shown whereby ≥ 1 is positive. The distribution of number of resistance-associated genes of β-lactam per sample among extensive antimicrobial use, limited antimicrobial use, and no antimicrobial use farms were shown.

The graph in Figure 5 shows the distribution of the number of positive genes for beta-lactam resistance per sample. Most Li-f and No-f carried 0–5 genes, whereas 80% of Ex-f carried ≥ 4 genes/sample. The mean of number of resistance genes in Ex-f (5.2 ± 2) was higher than Li-f (3.5 ± 1.8) and No-f (3.2 ± 1.6) (*p* < 0.05, One-way ANOVA).

Ten of 16 fluoroquinolone resistance associated genes were found. The gene *qnr*S was common in all farms (Figure 3B), however, copy number was higher in Ex-f than Li-f and No-f (Figure 4B). The *qnr*B1 was positive in 9.5% of Ex-f, 1.3% of Li-f and 0.7% of No-f. For *aac(6′)-lb-cr*, the percent positive was 6.3% of Ex-f and 0.7% of Li-f (Figure 3B).

The *gyr*A83L was the most frequent mutation in *gyr*A genes of *E. coli* and was higher in Ex-f (72.6%) compared to Li-f and No-f (55.3 and 38.7%, respectively). The less frequent *gyr*A87NY was detected in 4.7–8.4%. The point mutation at position Thr-86 in *gyrA* of *Campylobacter coli* was detected in 41.1% of Ex-f, 40.7% of Li-f and 28.7% of No-f (Figure 3B).

The common aminoglycoside resistance genes were *aac(3)-lVa*, *ant(2″)-Ia*, *aph(3′)-l*, and *aadA*, which were detected in all farms at 97.3–100% (Figure 3C). Although, a higher frequency of *aac(3)-lla* genes and *aac(6′)-lb* were detected in Ex-f (97.4 and 83.7%, respectively), they were high in Li-f and No-f as well (85.3 and 87.3% for *aac*(3)*-lla*; and 80.0 and 70.0% for *aac*(6′)*-lb*, respectively) (Figure 3C). The *arm*A was detected in 13/38 farms of Ex-f, whereas only one farm each of Li-f and No-f was positive.

The macrolide resistance marker gene *erm*B was present in all farms (Figure 3D). Ex-f had higher prevalence of *mph*A (77.9%) than Li-f and No-f (28.0%) (Figure 3D). The A2075G mutation in the 23S *rRNA* gene of *Campylobacter* spp. was also higher in Ex-f (77.4%), however Li-f and No-f also had substantial prevalence at 62.7 and 56.0%, respectively (Figure 3D).

The presence of *mcr-1* genes was 24.7% in Ex-f versus Li-f (9.3%) and No-f (6.0%), however, the frequency of *mcr-2* was not different in all farm types (27.9, 25.3, and 18.7%, respectively) (Figure 3D).

For chloramphenicol, tetracycline and trimethoprim-sulfonamides, *cml*A, *flo*R, *tet*A, *tet*B, *dfrA1*, *dfrA12*, *sul1*, *sul2* and *sul3* were commonly present in all farm categories with higher copy numbers in Ex-f (Figures 3, 4). Although these drugs were not used in farms, some genes were present in Ex-f more than Li-f and No-f such as *catA1* (31.6% vs. 16.0% and 12.0%, respectively) (Figure 3D) and *dfrA17* (53.2% vs. 24.7% and 16.0%, respectively) (Figure 3E).

### Confirmation of Resistance Phenotype

**FIGURE 6:**
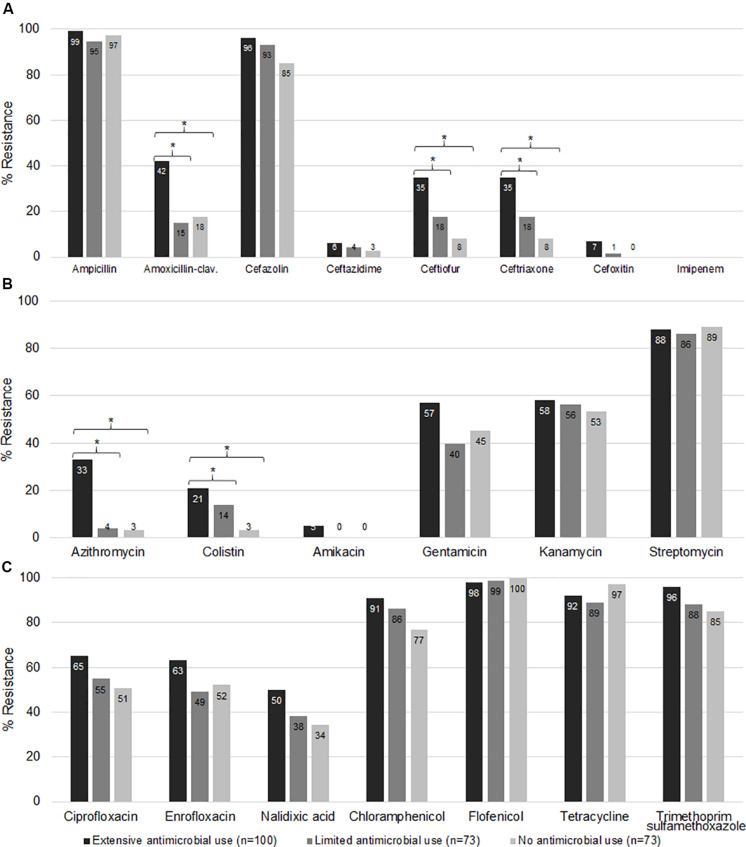
Phenotypic antimicrobial resistance. The *E. coli* isolated from fecal samples collected from three different farm categories were subjected to AST. The minimal inhibitory concentration (MIC) and zone diameter interpretative standard of CLSI-M100 Ed29 and CLSI-VET08 Ed4 (CLSI, 2018c, 2019) were used for interpretation. The percent resistance of β-lactam **(A)**, macrolides, polymyxins, and aminoglycosides **(B)**, quinolones, phenicols, tetracyclines, and trimethoprim-sulfonamides **(C)** among extensive antimicrobial use, limited antimicrobial use, and no antimicrobial use farms were shown. *, indicates *p* < 0.05 (Chi-square or Fisher’s exact test).

To audit and confirm the findings of the genotypic results, we also performed conventional susceptibility testing of *E. coli* isolates from the three different farm categories. The prevalence of resistance to amoxicillin-clavulanate, ceftiofur, ceftriaxone (Figure 6A), azithromycin, and colistin (Figure 6B) was higher for Ex-f than Li-f and No-f (*p* < 0.05, Chi-Square or Fisher’s Exact). On the other hand, resistance was not different for ampicillin, ceftazidime, cefoxitin (Figure 6A), amikacin, gentamicin, kanamycin, streptomycin (Figure 6B), ciprofloxacin, enrofloxacin, nalidixic acid, chloramphenicol, florfenicol, tetracycline, and trimethoprim-sulfamethoxazole (Figure 6C). There was a difference between Ex-f (96%) and No-f (85%) for cefazolin (Figure 6A). Lastly, there was no resistance observed for imipenem in any group (Figure 6A).

For beta lactam resistance, most isolates (*n* = 246) were resistant to ampicillin and cefazolin, whereas only 26.8, 22, 22, 4.5, and 3.3%, of the isolates were resistant to amoxicillin-clavulanate, ceftiofur, ceftriaxone, ceftazidime and cefoxitin, respectively. Resistance to amoxicillin-clavulanate (42%), ceftiofur (35%) and ceftriaxone (35%) in Ex-f were higher than Li-f and No-f (*p* < 0.05, Chi-Square or Fisher’s Exact) (Figure 6A).

The majority of isolates were resistant to chloramphenicol, florfenicol, tetracycline and trimethoprim-sulfamethoxazole at 85.4% (210/246), 98.8% (243/246), 93% (228/246), and 90% (222/246), respectively (Figure 6C).

### Timing of Antimicrobial Resistance

We then sought to examine the timing of AMR abundance in Ex-f for the genes of greatest relevance per Figure 3, namely *bla*_SHV_, *bla*_CTX–M1_, bla_CTX–M9_, *bla*_VEB_, *gyrA*83L-*E. coli*, *mphA*, 23S-*rRNA* 2075G-*Campylobacter* spp., and *mcr-1*. We performed a longitudinal study by collecting rectal swab samples from sows and their 3 weeks old weaning piglets, and these same pigs at 24 weeks of age. All samples were tested by AMR-TAC. The principal component analysis of all 66 resistance genes demonstrated an AMR profile among each group of subjects (Supplementary Figure S2). There was a significant difference in AMR genes between sows and piglets (*p* < 0.05, linear mixed model accounting for clustering within farm and within family), whereas there was not a significant difference between sows and 24-week pigs or between piglets and 24-week pigs.

**FIGURE 7:**
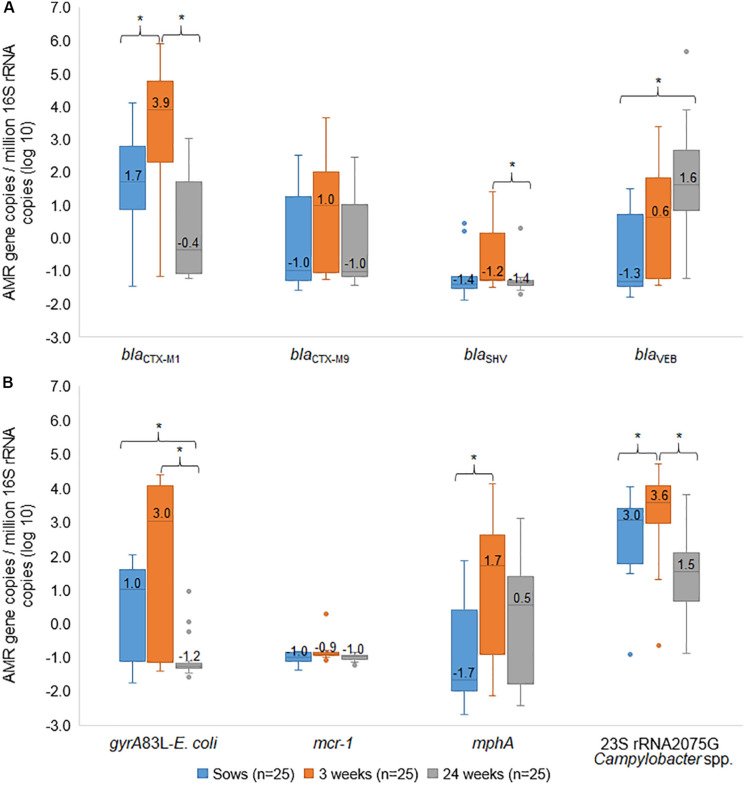
Box and whisker plots of resistance genes. The gene copies of *bla*_CTX–M1_, *bla*_CTX–M9_, *bla*_SHV_, *bla*_VEB_ **(A)**, *gyrA*83L-*E. coli*, *mcr-1*, *mphA*, and 23S *rRNA* 2075G-*Campylobacter* spp. **(B)** among sows, their 3 weeks old piglets and the same pigs at 24 weeks old were plotted. The horizontal line within the box indicates median, boundaries of the box indicate the 25th and 75th percentile, the whiskers indicate the highest and lowest values, the circle (o) indicates outlier. *, indicates *p* < 0.05, Friedman test between mean rank of groups.

There was not a consistent pattern of timing when antimicrobial resistance gene prevalence occurred. Some particular genes appeared to be frequent in sows and in their piglets (sow+/piglet+), suggesting acquisition from mother. These included *bla*_CTX–M1_; 21/25 (Supplementary Figures S3A,B), *gyrA*83L-*E. coli*; 14/25 (Supplementary Figures S3C,D), and 23S-*rRNA* 2075G-*Campylobacter* spp.; 24/25 (Supplementary Figures S3E,F). Interestingly, a high copy number of these genes was seen in piglets (log_10_ = 3.0–3.9) (*p* < 0.05, Friedman test) then turned to a low level in 24-wk pigs (log_10_ = −1.2–1.5) (*p* < 0.05, Friedman test) (Figures 7A,B). In addition, the linear mixed model confirmed these findings.

For *bla*_SHV_, most samples (15/25) were negative in all stages while 5/25 were positive in piglets, suggesting possibly early acquisition (Supplementary Figure S3G). This gene showed an increased copy number in piglets and then a decrease in 24-week pigs (*p* < 0.05, Friedman test) (Figure 7A), whereas no significant difference between sow and 24-week pigs by using cluster analysis (*p* > 0.05, linear mixed model). For *mcr*-1, the samples were negative in all stages except for one sample was positive in piglets (Supplementary Figure S3H).

Some particular genes appeared to suggest both acquisition from mother (sow+/piglets+), early acquisition after birth (sow-/piglets +), and later acquisition after weaning (piglets-/24-week pigs+). This included *bla*_CTX–M9_ (Supplementary Figures S4A–C), *bla*_VEB_ (Supplementary Figures S4D–F), and *mphA* (Supplementary Figures S4G–I). Copy numbers of *bla*_CTX–M9_ showed an increase in piglets but no significant difference from sows and their piglets (Figure 7A). For *bla*_VEB,_ copy numbers of this gene showed an increase from sow and higher in 24-week pigs (*p* < 0.05, Friedman test) (Figure 7A). By using the linear mixed model, significant lower *bla*_VEB_ was observed in sows than piglets and 24-week pigs, and 24-week pigs were higher than piglets (*p* < 0.05). For *mphA*, we noted the gene copy of *mphA* in piglets was significantly higher (*p* < 0.05, Friedman test and linear mixed model) than their sows (Figure 7B).

## Discussion

In this work, we detected AMR genes directly from fecal samples among three different swine management systems. The in-feed antimicrobial exposure (extensive use) farms, which utilized beta lactams and macrolides in feed, were distinct and had a higher prevalence of AMR genes than the limited and no antimicrobial use farms. Most notable was the increase in extended spectrum β-lactamase (ESBL) genes. ESBL producing Enterobacteriaceae (ESBL-PE) are of critical global public health importance (Maslikowska et al., 2016; Ahn et al., 2017; Flokas et al., 2017) and these pathogens have dramatically increased worldwide including in Thailand (Sawatwong et al., 2019).

The common ESBL genes observed in our study included *bla*_CTX–M1_ group, *bla*_CTX–M9_ group *bla*_OXA–1_ and *bla*_VEB_. Although, *bla*_CTX–M_ has been reported as the most prevalent ESBL gene type in swine farms, and *bla*_CTX–M1_ and *bla*_CTX–M9_ are common in Thailand, the high occurrence of *bla*_OXA–1_ and *bla*_VEB_ have not been reported from Thai agriculture farms (Hawkey, 2008; Suwantarat and Carroll, 2016; Lugsomya et al., 2018a). Detection of *bla*_VEB_ was first reported from Vietnamese patients and low prevalence or rare detection in human clinical isolates was published in many country (Poirel et al., 1999; Doi et al., 2017; Gay et al., 2018). Interestingly, in our study *bla*_VEB_ was enriched after weaning to 6 months of age, whereas *bla*_CTX–M1_ and *bla*_CTX–M9_ were reduced. The risk factors of *bla*_VEB_ enrichment in swine farm should be further explored, including ceftiofur use in swine. We speculate that the use of β-lactam in-feed is contributing to this ESBL development, since ESBL were less prevalent in Li-f and No-f. This has been reported previously (Fournier et al., 2019). That said, the mechanisms of AMR gene acquisition are clearly complex, as AMR genes in stool occur in the context of not only antibiotic exposure, but also the microbiome, weaning, and environmental exposure. Additionally, there are evidence of co-selection of AMR by using non-antimicrobial compounds such as biocide and heavy metals (Wales and Davies, 2015; Cheng et al., 2019). Furthermore, a study of Danish pig farms concluded that the effect of antimicrobial exposure on the levels of AMR genes was complex and unique for each individual AMR gene (Birkegard et al., 2017).

In addition to ESBL genes, the in-feed antimicrobial using farms also had a higher rate of other AMR genes, even to antimicrobial classes that were not present in feed. For instance, we saw increases in certain aminoglycoside resistance genes, even though they were not utilized by the in-feed farms. These genes are transmissible, encoded on conjugative plasmids, and often linked to resistance to other antimicrobials (Galimand et al., 2003; Evershed et al., 2009; Ramirez and Tolmasky, 2010). It has been noted previously that routine use of unrelated antimicrobial agents can increase resistance to aminoglycosides (Lugsomya et al., 2018a). There were other antimicrobial classes which were not utilized in any farms (trimethoprim-sulfonamides, tetracyclines, and phenicols) but the in-feed antimicrobial use farms showed a higher gene copy than the other two groups. One possible explanation for this phenomena could be co-selection or co-transfer of gene cassettes on integrons (Bischoff et al., 2005; Evershed et al., 2009; Domingues et al., 2012). There are reported integrons found in β-lactamase positive isolates (Yaqoob et al., 2011). Common gene cassettes of integrons contain *sul*, *dfrA*, and *aadA* genes which confer resistance to sulfonamides, trimethoprim and streptomycin (Domingues et al., 2015). The co-transference of resistance to florfenicol and oxytetracycline with integron was also reported previously (Dominguez et al., 2019).

Colistin was not used in this study but colistin resistant isolates of *E. coli* and *mcr-*1 and *mcr-2* genes were found. Notably, these genes were also lower in Li-f and No-f. Recently, *E. coli* carrying both *mcr-1* and ESBL in the plasmid in swine in China have been reported (Malhotra-Kumar et al., 2016; Ji et al., 2019; Lee et al., 2020).

Among quinolone resistance, *qnr*S as well as *aac*(6′)-*Ib-cr* positive plasmids have been detected in many countries. Our study’s occurrence of *aac-*(6′)*-lb-cr* gene in swine farms was relatively low (13/490, 2.65%) compare to previously reported in human clinical *E. coli* isolates (Hassan et al., 2012; Soundararajan et al., 2016). In contrast, occurrence of *qnr*S was high in all farm type including No-f. The co-carried of *qnr*S gene in the same mobile genetic element with *bla*_TEM_ and ESBL gene have been reported (Kehrenberg et al., 2006; Jiang et al., 2012).

For macrolides, *erm* gene was common and widespread (Bolinger and Kathariou, 2017). However, the *mph*A gene was increased in the Ex-f which could be concerning. The *mph*A gene is commonly found on mobile genetic elements, and is a phosphotransferase that inactivates macrolides, and could have been induced by tylosin in swine. This gene has been reported on plasmids that encode CTX-M as well (Fyfe et al., 2016). A high prevalence of macrolide resistant *Campylobacter* spp. was present in this study. The A2075G substitutions in 23S rRNA gene is the most common mutation conferring macrolide resistance and was seen at 77%.

Our genotypic results were generally supported by phenotypic AST, as was expected based on our earlier work comparing genotypic results on stool with phenotypic results on fecal *E. coli* isolates (Pholwat et al., 2019). Of note, the phenotypic resistance rate for most antimicrobial agents in our study was quite high, generally higher than previously reported in Thailand from swine farms (Nhung et al., 2016; Lugsomya et al., 2018b). This trend should be monitored in the future.

The results from longitudinal study suggested that AMR acquisition may occur both from mother and after weaning, presumably from other sources in the environment. The quantity of AMR gene was likely higher in 3 weeks old weaning piglets than their sows and 24 weeks old at finishing stage which consistent with the higher number of *E. coli* these could be explained by diversity of microbiome which shift overtime of production stages. There was previously study showed *E. coli* was abundant present during the lactation stage and persisted till the end of nursery phase before phasing out (Wang et al., 2019). Our AMR gene targets were largely specific to Enterobacteriaceae, therefore the abundant of *E. coli* in nursery phase increase probability to detect AMR gene compare to the less abundant of *E. coli* in the older pigs. This consistent with a reported previously that aminoglycoside resistant and ESBL producing *E. coli* are more commonly found in the nursery and growing periods of pig production (Lugsomya et al., 2018b).

While our findings support the association of AMR with non-therapeutic antimicrobial (NTA) use in food producing animal, as has been reported previously (Lugsomya et al., 2018a), this link between antimicrobial use in animals and AMR in human still receives debate. Some studies have suggested a link between animal and humans for transmissions of certain *Staphylococcus aureus* clones (Ward et al., 2014). By contrast, studies of *Salmonella Typhimurium* DT104 (DT104) showed animals and humans have distinguishable DT104 communities, suggesting that animal populations may not be a common source of resistant *Salmonella* (Mather et al., 2012, 2013). We propose that larger scale, ideally quantitative, surveillance of human, environmental, and animal sources for AMR genes will be helpful to understand where the largest burden of AMR genes derives. Our work shows that direct molecular detection of AMR genes through this approach is promising, and points to NTA use in food producing animal as an important component of AMR, particularly ESBL.

## Data Availability Statement

The raw data supporting the conclusions of this article was made available in Supplementary Tables S1, S2.

## Ethics Statement

The animal study was reviewed and approved by the Siriraj Animal Care and Use Committees, Faculty of Medicine Siriraj Hospital, Mahidol University. Written informed consent for participation was not obtained from the owners because the study involve only obtaining fecal samples from animal and the owners agree to participate the study with no written informed consent required.

## Author Contributions

SP performed the experiments, analyzed the data, and prepared the draft of the manuscript. TP and RC contributed to accessing and providing resources. ER contributed to the statistical analysis. IT, PR, JL, and MT contributed to the study design and data analysis supervision. EH and SF contributed to the conception and design of the study, funding acquisition, supervision, and wrote the manuscript. All authors contributed to manuscript revision, read, and approved the submitted version.

## Conflict of Interest

TP was employed by Charoen Pokphand Foods PCL which provided support in the form of salaries for authors TP and swine fecal sample materials but did not have any additional role in the study design, data collection and analysis, preparation of the manuscript, or decision to publish. The remaining authors declare that the research was conducted in the absence of any commercial or financial relationships that could be construed as a potential conflict of interest.
