# Supplementary figures and images for: Antimicrobial Resistance in Swine Fecal Specimens Across Different Farm Management Systems
Source: Front Microbiol. 2020 Jun 17;11:1238. doi: 10.3389/fmicb.2020.01238 (PMC7311580; doi:10.3389/fmicb.2020.01238)

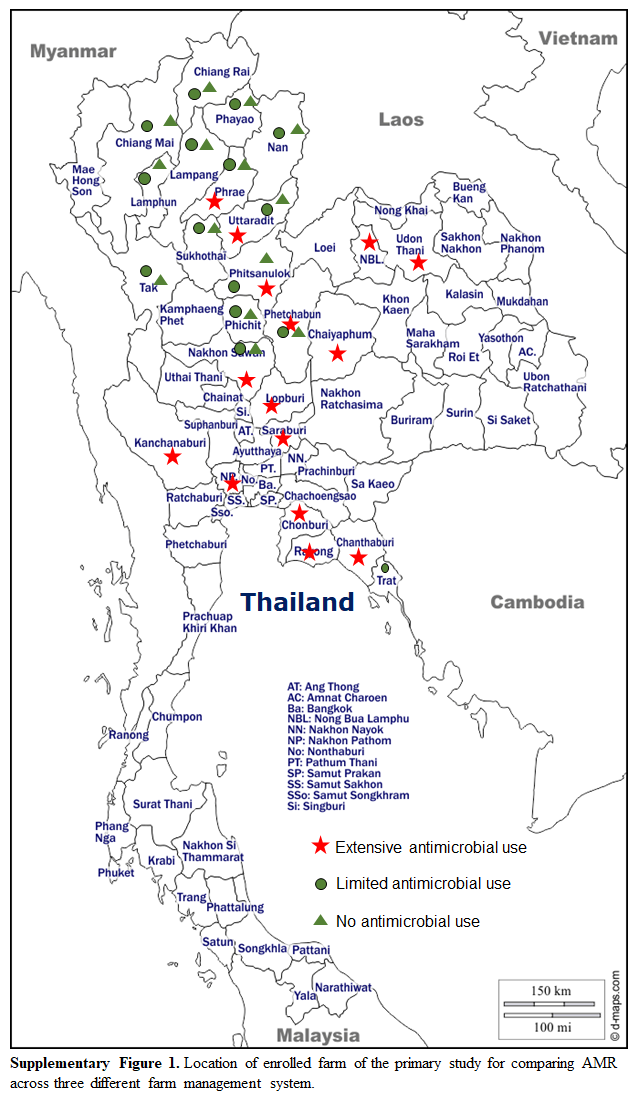

Supplement: Supplementary file 1 [file Image_1.TIF]

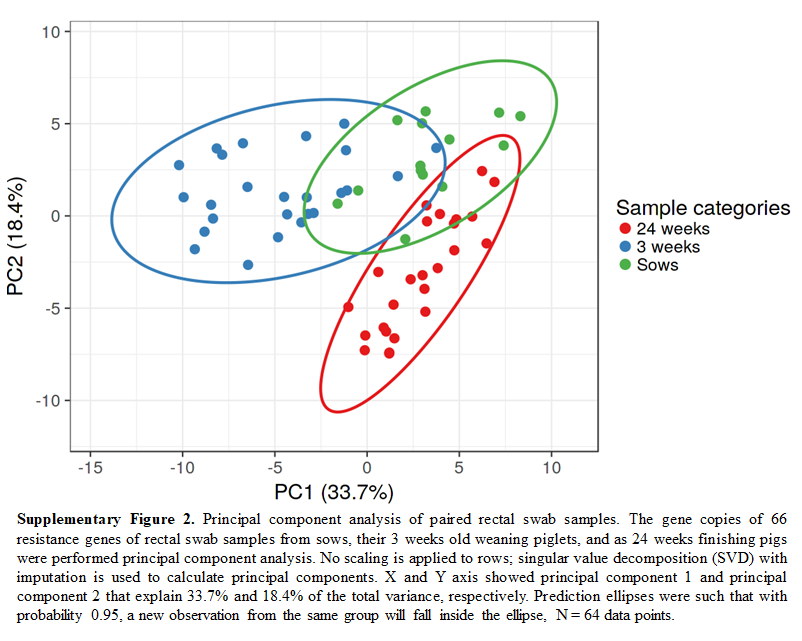

Supplement: Supplementary file 2 [file Image_2.TIF]

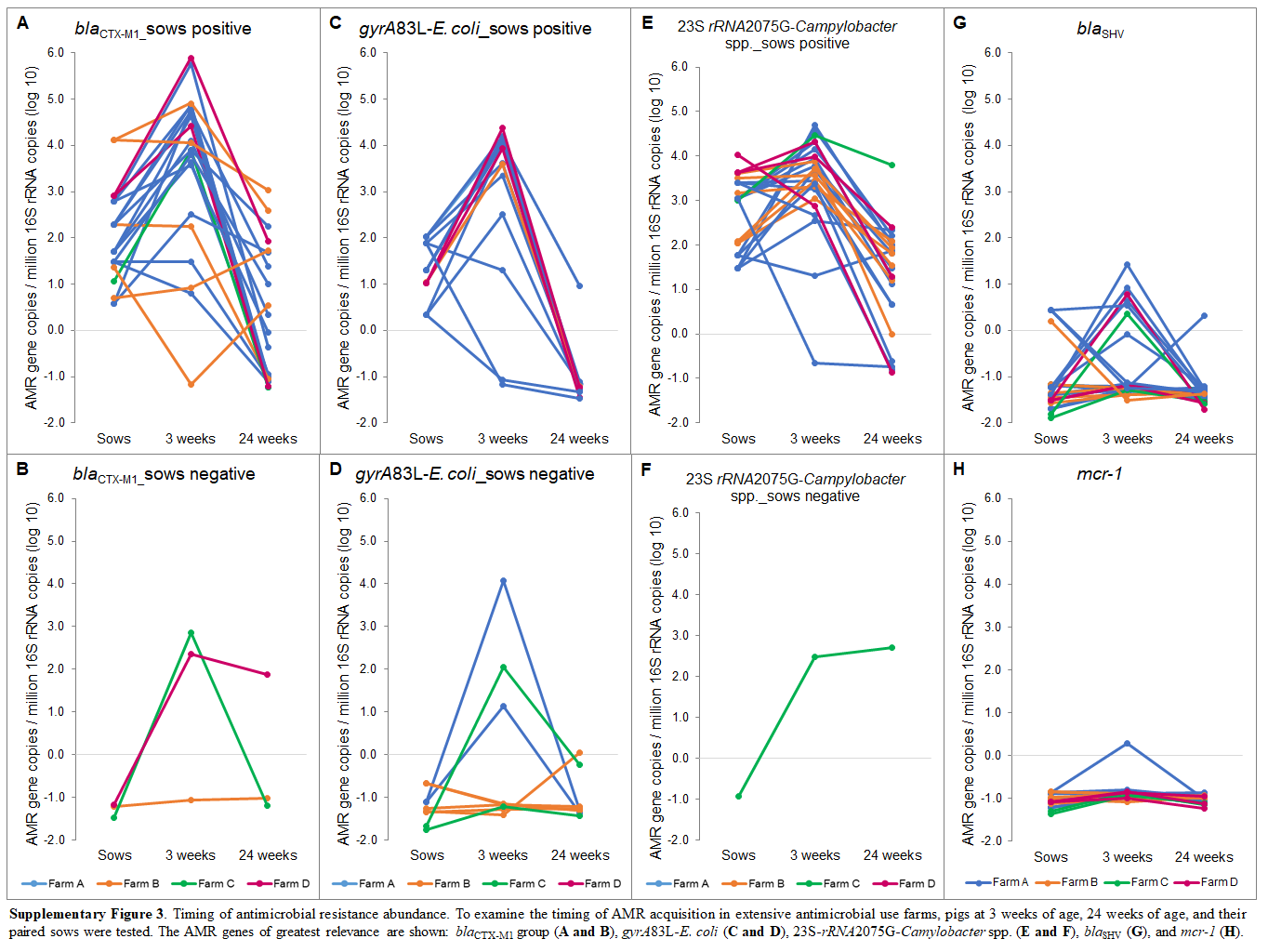

Supplement: Supplementary file 3 [file Image_3.TIF]

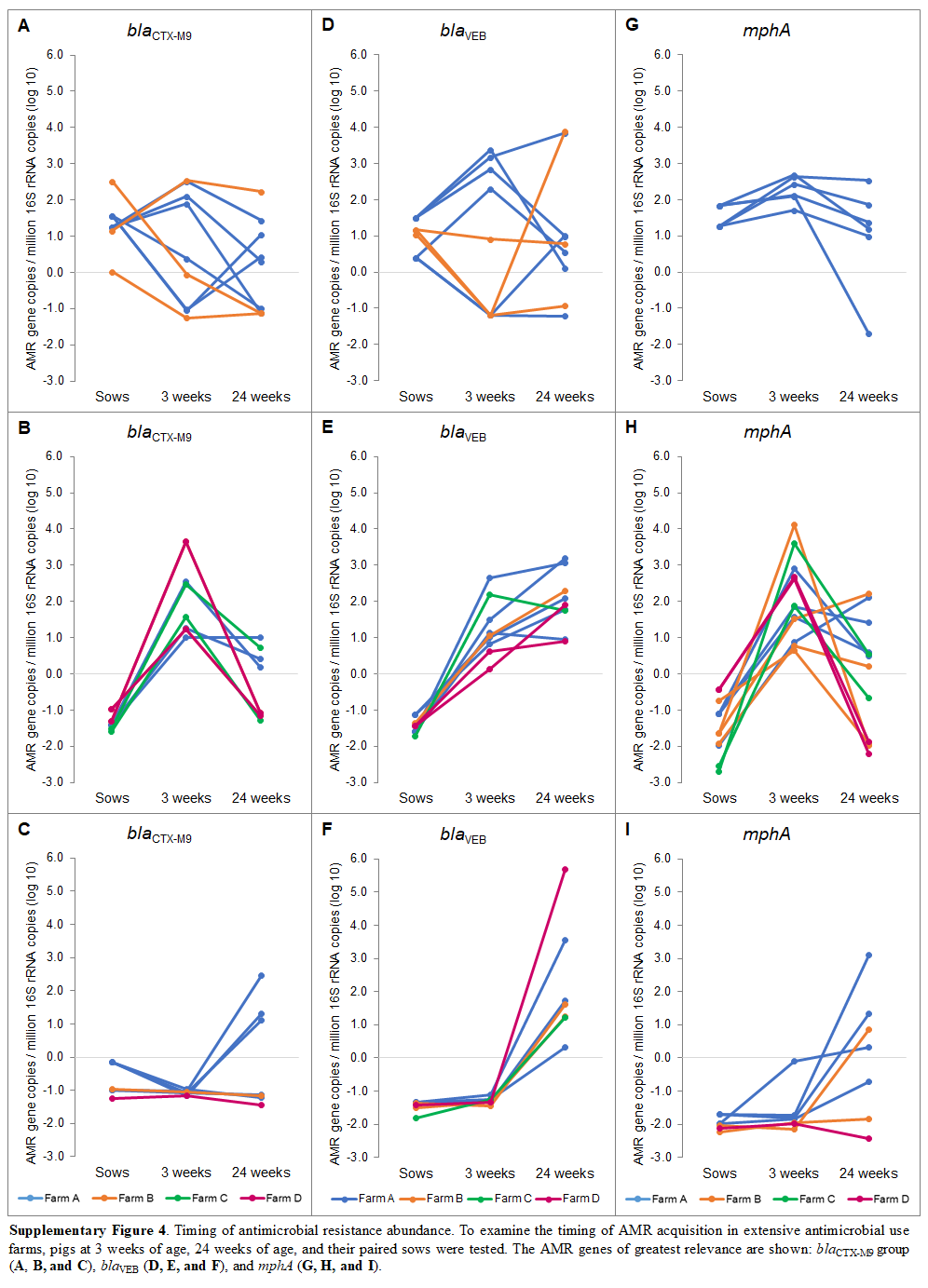

Supplement: Supplementary file 4 [file Image_4.TIF]
